# Structural and functional characterization of the CAP domain of pathogen-related yeast 1 (Pry1) protein

**DOI:** 10.1038/srep28838

**Published:** 2016-06-27

**Authors:** Rabih Darwiche, Alan Kelleher, Elissa M. Hudspeth, Roger Schneiter, Oluwatoyin A. Asojo

**Affiliations:** 1 Division of Biochemistry, Department of Biology, University of Fribourg Chemin du Musée 10 CH 1700 Fribourg Switzerland; 2 National School of Tropical Medicine, Baylor College of Medicine Houston TX 77030 USA

## Abstract

The production, crystal structure, and functional characterization of the C-terminal cysteine-rich secretory protein/antigen 5/pathogenesis related-1 (CAP) domain of pathogen-related yeast protein-1 (Pry1) from *Saccharomyces cerevisiae* is presented. The CAP domain of Pry1 (Pry1CAP) is functional *in vivo* as its expression restores cholesterol export to yeast mutants lacking endogenous Pry1 and Pry2. Recombinant Pry1CAP forms dimers in solution, is sufficient for *in vitro* cholesterol binding, and has comparable binding properties as full-length Pry1. Two crystal structures of Pry1CAP are reported, one with Mg^2+^ coordinated to the conserved CAP tetrad (His208, Glu215, Glu233 and His250) in spacegroup *I*4_1_ and the other without divalent cations in spacegroup *P*6_1_22. The latter structure contains four 1,4-dioxane molecules from the crystallization solution, one of which sits in the cholesterol binding site. Both structures reveal that the divalent cation and cholesterol binding sites are connected upon dimerization, providing a structural basis for the observed Mg^2+^-dependent sterol binding by Pry1.

Members of the eukaryotic CAP (cysteine-rich secretory protein/antigen 5/pathogenesis related-1) or SCP/TAPS (Sperm-coating protein/Tpx/antigen 5/pathogenesis related-1/Sc7) superfamily have a wide range of physiological activities including sperm maturation, fertilization, fungal virulence, cellular defense, and immune evasion^1^,^2^,^3^,^4^,^5^,^6^. However, the underlying mechanisms by which CAP proteins perform these seemingly unrelated functions remain poorly understood. CAP proteins contain a CAP or SCP domain, named after mammalian CAP proteins characterized in semen and seminal fluid^7^,^8^,^9^,^10^. The CAP domain (NCBI domain cd00168 or Pfam PF00188) is an ~15 kDa cysteine-rich domain with limited (<38%) sequence identity^1^,^2^,^3^,^4^. Several CAP protein structures have been reported and they all contain a conserved alpha-beta-alpha sandwich domain^8^,^11^,^12^,^13^,^14^,^15^,^16^,^17^,^18^,^19^,^20^. The CAP domains differ in the lengths of their strands and helices and ~47% of the domain is composed of loops, making it difficult to accurately predict their structure^11^,^12^,^13^. The prototypical CAP domain has a central cavity characterized by the cavity tetrad, formed by four key residues from the four CAP motifs: His from CAP1, Glu from CAP2, His from CAP3, and Glu from CAP4 and these tetrad residues bind divalent cations including zinc and magnesium^8^,^13^,^21^,^22^. CAP1 and CAP2 are defined in the PROSITE database (http://www.expasy.ch/prosite) as CRISP motifs, while CAP3 and CAP4 are additional CAP motifs defined by Gibbs and colleagues^8^.

CAP proteins bind lipids; for example Tablysin-15 from a blood-feeding arthropod is an anti-inflammatory scavenger of eicosanoids with a hydrophobic channel that binds leukotrienes with sub-micromolar affinities^19^. Structures revealing how palmitate and other lipids bind to this hydrophobic channel have been reported^19^. Additionally, GLIPR2/GAPR-1 which is highly over-expressed in glioblastoma multiforme, binds to the surface of liposomes containing negatively charged lipids^23^,^24^. Structures showing how GAPR-1 binds up to three phosphatidylinositol molecules strongly enough to resist denaturation or organic solvent extraction have been reported^23^,^24^. Our previous studies revealed that *Saccharomyces cerevisiae* CAP proteins are required for the export of sterols *in vivo* and they bind cholesterol *in vitro*^25^,^26^.

The *Saccharomyces cerevisiae* genome encodes three CAP proteins known as Pathogen Related in Yeast (Pry1–3). Pry1 and Pry2 are secreted glycoproteins and Pry3 is associated with the yeast cell wall^25^. The sterol binding and export properties of these proteins are localized to the CAP domain, which is sufficient to rescue sterol export properties of cells lacking endogenous Pry proteins^25^,^26^. Computational modeling suggests that sterol binding to Pry1 occurs through displacement of a flexible loop, the caveolin binding motif (CBM)^25^,^26^. While point mutations within the CBM abrogated sterol binding and export, mutations of residues located outside the CBM including highly conserved putative catalytic residues have minimal effect on lipid binding and sterol export^23^. These studies defined the CBM as a crucial motif for lipid binding *in vitro* and sterol export *in vivo*^22^,^23^. Furthermore, the expression of heterologous CAP proteins rescues the block in sterol export of yeast mutants lacking Pry function, indicating that sterol export is a conserved function of CAP proteins from different species^26^,^27^. As part of our ongoing efforts to characterize the lipid binding properties of this ubiquitous superfamily of proteins^6^,^11^,^13^,^25^,^26^,^27^,^28^, we present the structural and functional characterization of the CAP domain of Pry1 (Pry1CAP) from *Saccharomyces cerevisiae*.

## Results

### Pry1CAP forms dimers in solution

**Figure 1:**
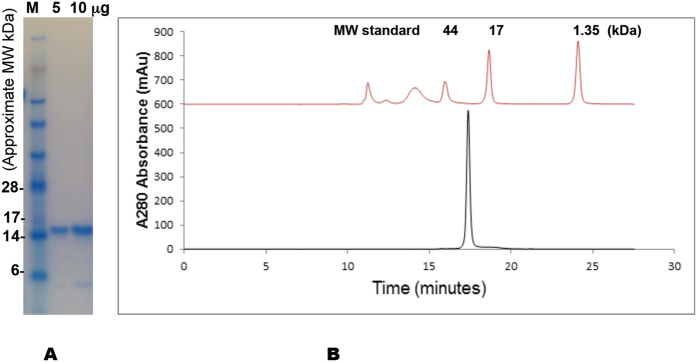
Pry1CAP purification and size exclusion chromatography. (**A**) The electrophoretic mobility of Pry1CAP on a reducing SDS Page gel reveals a monomeric weight of ~15 kDa, (**B**) Pry1CAP elutes as an ~31 kDa dimer from a size exclusion column.

Pry1CAP was overexpressed in *Pichia pastoris* with a yield of approximately 500 mg of ~99% pure protein from a 1 L shake flask culture. Pry1CAP migrates on a reducing Coomassie stained SDS PAGE gel at ~16 kDa (Fig. 1A). As was observed for some CAP proteins including Glipr-1, GAPR-1 and *Na*-ASP-2, Pry1CAP crystallizes with a monomer in the assymetric unit while dimerizing in solution^8^,^11^,^12^, eluting from a size exclusion column as a single sharp peak, with a molecular mass of 30.8 kDa, which is twice the theoretical molecular weight of 15.9 kDa (Fig. 1B).

### Pry1CAP binds and exports cholesterol

**Figure 2:**
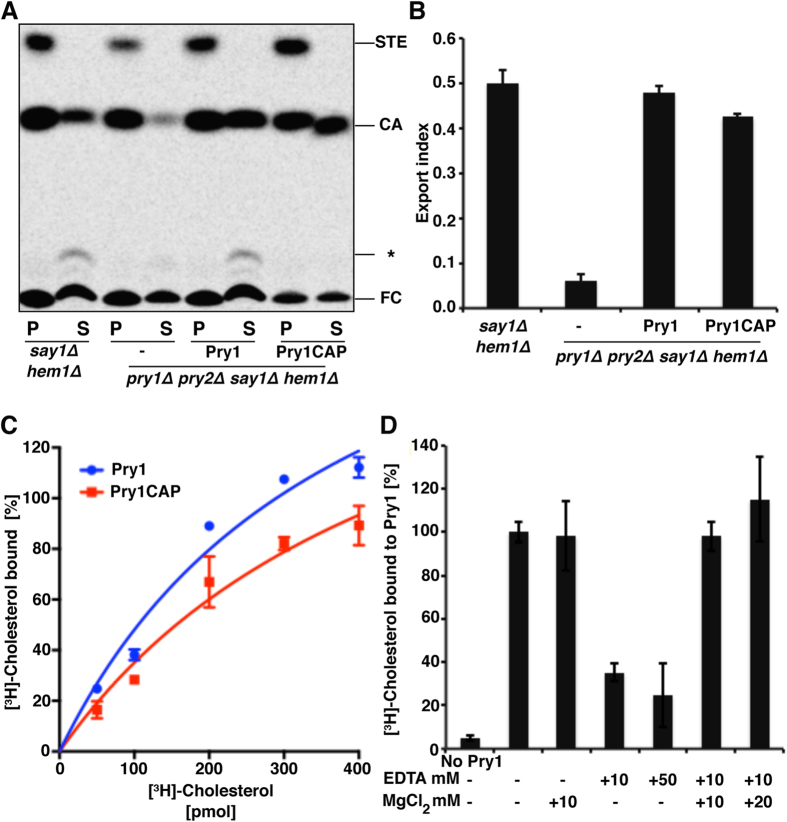
The CAP domain of Pry1 rescues the defect in cholesterol acetate export of the yeast *pry1Δ pry2Δ* double mutant *in vivo* and binds cholesterol *in vitro.* (**A**) Expression of Pry1CAP complements the sterol-export defect of yeast cells lacking their endogenous CAP proteins. Levels of lipids from Say1 and Heme deficient cells (*say1Δ hem1Δ*) containing either an empty plasmid (−) or a plasmid with Pry1 or Pry1CAP in the cell pellet (P) and the culture supernatant (S) after separation by thin-layer chromatography. The positions of free cholesterol (FC), cholesteryl acetate (CA), steryl esters (STE), and other cholesterol derivatives (*) are indicated. (**B**) Quantification of the levels of exported CA from yeast cells lacking their endogenous CAP proteins compared with levels from cells complemented with Pry1 or Pry1CAP. (**C**) Pry1CAP binds cholesterol *in vitro* similarly to full-length Pry1. (**D**) Sterol binding is Mg^2+^-dependent.

Pry1CAP used in these studies is functional for cholesteryl acetate export *in vivo* because a plasmid encoding Pry1CAP complemented the cholesteryl acetate export defect of mutant yeast cells that lacked endogenous Pry1 and Pry2 (Fig. 2A). The efficiency of cholesteryl acetate export by Pry1CAP was comparable to that of full-length Pry1 as indicated by the similarity of the export indices (Fig. 2B). Addition of an increasing amount of [^3^H]-cholesterol resulted in a concentration dependent and saturable binding of cholesterol to Pry1CAP protein. *In vitro*, Pry1CAP displayed saturation binding kinetics with an apparent *K*_*d*_ of 2.08 ± 0.07 μM, which is comparable to cholesterol binding by full-length Pry1 (*K*_*d*_ of 1.25 ± 0.42 μM) (Fig. 2C). The saturable binding observed by titration of the ligand is not due to limited solubility of cholesterol, as indicated by the control experiment in which the *in vitro* affinity of Pry1 to cholesterol was measured by increasing the concentrations of the purified protein (0–500 pmol) rather than increasing the concentration of the radioligand. This results in a saturable binding curve with an apparent *K*_*d*_ of 0.87 ± 0.18, which is very similar to the *K*_*d*_ obtained by titrating the ligand (*K*_*d*_ of 1.25 ± 0.42 μM). Furthermore, cholesterol binding by Pry1CAP is inhibited by EDTA and adding magnesium ions restores sterol binding, indicating that magnesium is important for sterol binding by Pry1CAP (Fig. 2D).

### Overall Structure of Pry1CAP

**Figure 3:**
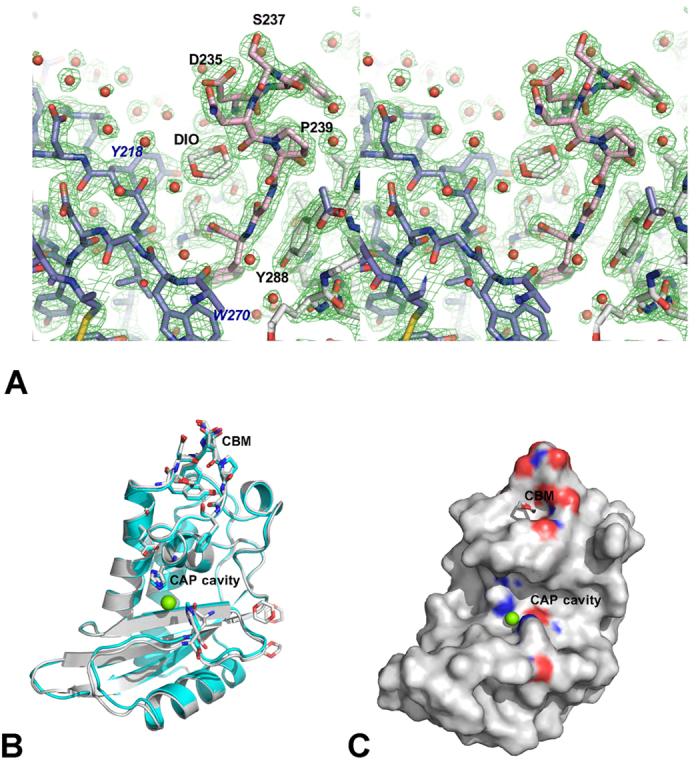
The Pry1CAP structure. (**A**) Stereoview of the 1,4-dioxane molecule in a 2*F*_o_ − *F*_c_ electron-density map (green) contoured at 1.5 σ bound in the caveolin-binding motif (CBM) cavity of Pry1CAP. Main chain residues are colored gray, the CBM residues are shown in pink stick, residues from a symmetry related monomer are colored in blue, and water molecules are shown as red spheres. (**B**) Superposed structures of the complexes Pry1CAP with Mg^2+^ ion (green) and 1,4-dioxane (gray). (**C**) Location of the CBM and CAP tetrad on Pry1CAP monomer surface representation.

**Figure 4:**
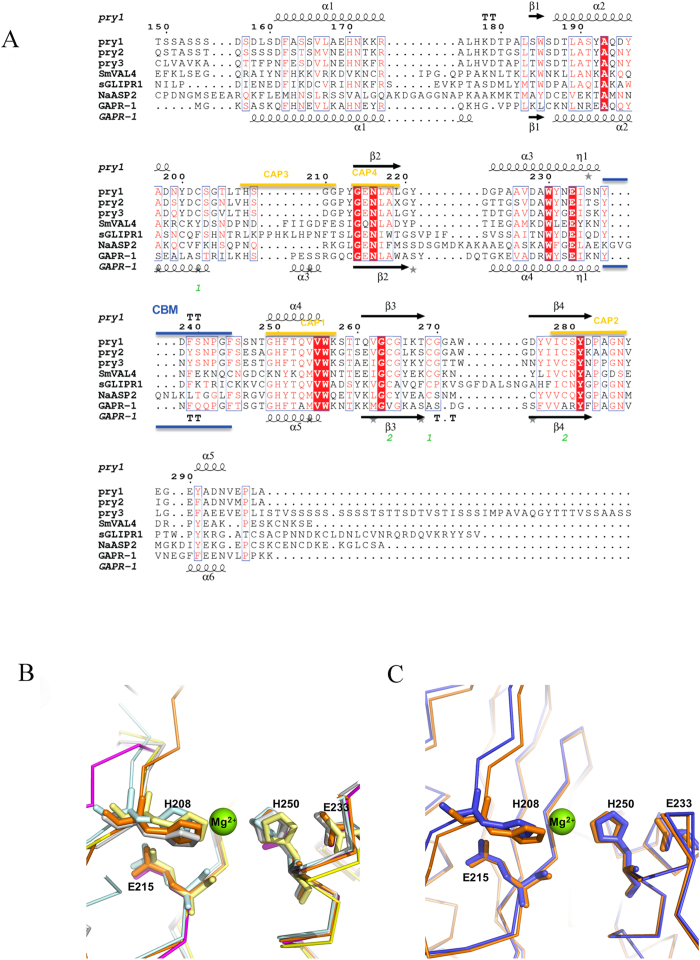
CAP protein motifs. (**A**) Structural features of Pry1CAP and primary sequence alignment with selected representative CAP proteins. This figure was generated with ESPript^48^. The different secondary structure elements shown are alpha helices as large squiggles labelled (α), 3_10_-helices as small squiggles labelled (η), beta strands as arrows (β), and beta turns (TT). Identical residues are shown in white on red background, and conserved residues in red. The locations of the cysteine residues involved in disulfide bonds are numbered in green. CAP motifs are highlighted in gold. The representative CAP structures are *Na*-ASP-2 (PDB entry 1u53), SmVAL4 (PDB entry 4p27), GAPR-1 (PDB entry 1smb), and sGLIPR1 (PDB entry 3q2r). (**B**) The CAP tetrad residues of Pry1CAP (PDB entry 5jys) superimpose with other CAP structures. CAP structures are colored as follows Pry1CAP (orange), SmVAL4 (magenta), GAPR-1 (cyan), sGLIPR1 (gray) and *Na*-ASP-2 (yellow). (**C**) The superposed divalent cation binding site of Pry1CAP with Mg^2+^ (PDB entry 5jys, orange) and without Mg^2+^ (PDB entry 5ete, blue). The numbers correspond to those of Pry1CAP and the magnesium ion is shown as green sphere.

Two Pry1CAP structures solved by molecular replacement with a monomer in the assymetric unit are reported and their atomic coordinate and structure factors have been deposited in the protein data bank under accession number 5ETE and 5JYS, for the 1,4-dioxane and Mg^2+^ complex, respectively. Both Pry1CAP structures are very similar with an rmsd of all atoms of 0.318 Å. In both structures amino-terminal amino acids residues Ser151 through Ser157, which contains two predicted O-glycosylated sites, are disordered. The overall monomer surface area for Pry1CAP is 7125 Å^2^ and its topology is an alpha-beta-alpha sandwich made up of 6 helices, 2 disulfide bonds, 12 beta turns and 1 beta bulge (Fig. 3B,C). The overall structure of Pry1CAP is 16.9% strand, 33.1% alpha-helix, 2.1% 3–10 helix and 47.9% loop with longer loops than predicted based on other CAP structures (Fig. 4A). The 3–10 helix is involved in the alpha-beta-alpha sandwich and was previously observed in SmVAL4 but not in any of the other representative CAP protein structures.

### Divalent cation binding

Pry1CAP has a large central (1638 Å^3^ volume) cavity, which contains the tetrad that binds divalent cation other CAP protein^11^,^14^,^19^,^21^,^23^,^29^, and this cavity is distinct from the CBM cavity (Figs 3C and 4A). The CAP cavity tetrad residues of Pry1CAP (His208, Glu215, Glu233 and His247) superpose well with the corresponding residues from representative CAP proteins^8^,^11^,^13^,^29^ (Fig. 4B). In one Pry1CAP structure, a magnesium ion from the crystallization solution is coordinated by the tetrad which results in a slight shift in the histidines when compared to the structure without the divalent cation (Fig. 4C).

## Discussion

### 1,4-Dioxane binds to the cholesterol binding site

Electron density for four 1,4-dioxane molecules were observed in the initial molecular replacement 2*F*_o_ − *F*_c_ maps when dioxane was present in the crystallization solution; this density is absent in the Mg^2+^ structure, which does not have 1,4-dioxane in the crystallization solution. Density corresponding to one 1,4-dioxane molecule was observed in the 194 Å^3^ volume cavity created by the CBM, confirming for the first time the ability of the cholesterol binding site of Pry1 to bind a ligand.

Since a 1,4-dioxane molecule binds to the CBM cavity, and the CBM is verified to be important for cholesterol binding^26^, the effect of 1,4-dioxane on cholesterol binding was tested. 1,4-dioxane inhibits *in vitro* binding of [^3^H]-cholesterol to Pry1CAP in a dose dependent manner (Figure S.1). However, the inhibition was only observed at relatively high concentrations of 1,4-dioxane, and is possibly due to the increased hydrophobicity of the 1,4-dioxane containing solvent compared to the aqueous buffer. Interestingly, 1,4-dioxane does not bind to other lipid binding sites notably the palmitate binding site of Tablysin-15^19^ or phosphatidylinositol binding sites of GAPR-1^23^,^24^. The presence of dioxane in the crystallization solution was incompatible with the formation of complexes of Pry1CAP with cholesterol or palmitate and all co-crystallization and soaking experiments yielded crystals with dioxane only. While cholesterol is virtually insoluble in water, it is soluble in 1,4-dioxane, and 1,4-dioxane is a major component of the CryoSol kit that is used for co-crystallizing proteins with hydrophobic ligands^30^,^31^,^32^. Efforts are underway to identify either a sterol solubilizing agent that is suitable for co-crystallization or a crystallization condition that is compatible with sterol binding.

### Comparison of Pry1CAP with other CAP proteins

Using PDBFold, GAPR-1 with bound inositol hexakisphosphate (PDB entry 4aiw)^23^, and the apo structure of the same protein (PDB entry 1smb)^14^ were identified as the most similar structures to that of Pry1CAP. The second best score was that of VAL4 from *Schistosoma mansoni* (SmVAL4), which lacks the prototypical CAP cavity (PDB entry 4p27)^27^. This is followed by the NMR structure of a plant CAP protein (P14a, PDB entry 1cfe)^15^; crystal structures of the hookworm CAP protein *Na*-ASP-2, (PDB entries 4ly5, 4nui, 4nuo, 4nuk)^13^,^22^; the structures of human glioma pathogenesis related protein (sGLIPR1 PDB entry 3q2r)^13^; and structures of snake venom CRISPs notably pseudechetoxin (PDB entry 2dda)^29^. All these proteins share under 35% sequence identity with Pry1CAP and the greatest differences between representative CAP protein structures are in loop regions as well as in the length of helices and strands (Fig. 4A). The flexible regions in CAP proteins are important for ligand binding and make up ~47% of the structure. Therefore, each new CAP protein structure offers information that cannot be generated simply by homology modeling.

### The CAP tetrad and CBM cavities are connected in the dimer

**Figure 5:**
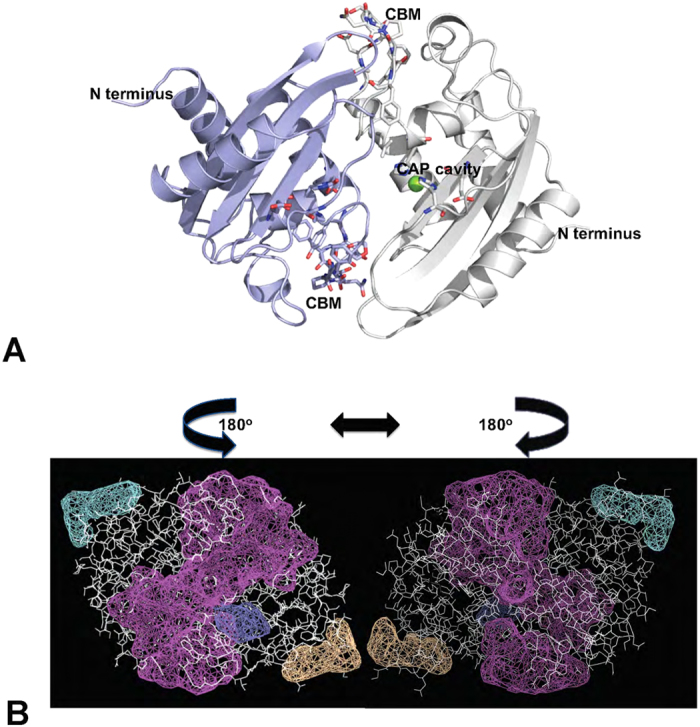
Pry1CAP dimer. (**A**) Ribbon diagram of the crystallographic dimer with one monomer colored blue and the other gray. (**B**) A large central cleft (purple) wraps around the protein (white sticks) connecting the CBM and CAP tetrad sites of both monomers, the left panel is the same view as Fig. 5A while the right panel represents a 180° rotation around the 2-fold axis. Additional clefts in the structure are shown as mesh in other colors.

The observation that EDTA inhibits cholesterol binding and that addition of Mg^2+^ restores cholesterol binding by Pry1 is surprising since the cholesterol binding CBM and the CAP tetrad that binds Mg^2+^ are located at distinct sites in the Pry1CAP monomer. Furthermore, our previous studies identified SmVAL4, a CAP protein lacking the CAP tetrad as an effective sterol binder and exporter^27^. Mg^2+^ dependent sterol binding by Pry1CAP requires the interaction of both binding sites, likely occuring *via* dimerization, because Pry1CAP forms dimers in solution, and both crystal structures have a crystallographic dimer in which the CBM and CAP tetrad are connected within a large 7063 Å^3^ volume cleft (Fig. 5). The same dimer was observed in two different structures of Pry1CAP, that were obtained from different crystallization conditions with different spacegroups and crystal morphologies. Incidentally, the cavities of the two covalently linked CAP domains of *Na*-ASP-1^12^ are connected by a large cleft and prior to this study a dimer that allowed the connection of the CAP tetrad and lipid binding cavities of single CAP domain proteins had not been identified^8^,^11^,^12^. Beyond affecting sterol binding, the implications of having such a large interconnected cleft are undefined; however, the cavity volume is large enough to bind other ligands, such as peptides, which may be relevant for some observed functions of other eukaryotic CAP proteins, for example, the serine protease activity of the cone snail CAP protein Tex31^33^ and the ability of the hookworm NIF to inhibit neutrophils^34^.

## Conclusions

Two crystal structures of the CAP domain of pathogen-related yeast protein from *Saccharomyces cerevisiae*, Pry1CAP, are presented. Both structures reveal that the cholesterol binding CBM is large enough for cholesterol binding. In one structure a 1,4-dioxane molecule from the crystallization mixture occupies the CBM cavity confirming its ability to bind hydrophobic ligands. In the second structure Mg^2+^ is coordinated by the CAP cavity tetrad residues. Pry1CAP is functional in cholesterol export *in vivo* and binds cholesterol *in vitro* with comparable affinity to full length Pry1. Interestingly, cholesterol binding by Pry1CAP is inhibited by EDTA and restored by the addition of Mg^2+^ indicating that presence of the divalent cation is important for sterol binding. The cholesterol and Mg^2+^ binding sites are distinct and unconnected in the Pry1CAP monomer. Pry1CAP is a dimer in solution and the cholesterol and Mg^2+^ binding sites are connected by a large cleft in a crystallographic dimer, providing a structural basis for Mg^2+^ -dependent sterol binding by Pry1CAP.

## Methods

### Recombinant protein expression and purification of Pry1CAP

The carboxyl terminal CAP domain (corresponding to amino acid residues Ser151 through Ala296) of Pry1 from *Saccharomyces cerevisiae* was amplified by PCR and ligated into pPICZαA vector using the XhoI and XbaI sites. After linearization, the vector was transformed into *Pichia pastoris* strain X33. The transformants were selected on zeocin-resistant YPD plates and verified by PCR amplification using pPICZαA vector flanking primers (α-factor and 3′AOX1). Ten colonies with the right insert were picked and screened for induction of recombinant Pry1CAP protein with 0.5% methanol at 30 °C for 72 hours. The highest expressing colony was chosen for recombinant protein expression and purification as previously described for SmVAL4^27^. Full length Pry1 was produced similarly to Pry1CAP.

### Size-exclusion chromatography (SEC)

For SEC experiments, 20 μg of Pry1CAP was injected onto a Yarra SEC-2000 column (Phenomenex, Torrance, CA) at flow-rate of 0.5 ml/min with a Shimadzu Prominence series HPLC (Kyoto, Japan) using PBS pH 7.4 as the mobile phase. The elution was monitored with a photo diode array detector (Shimadzu). The system was calibrated using Bio-Rad gel filtration standard (Hercules, CA) consisting of proteins with molecular weights of 670, 158, 44, 17 and 1.35 kDa. Data analysis was performed on the 280 nm wavelength extracted chromatograph using Shimadzu LCsolution version 1.25. Experiments were conducted in triplicate.

### Crystallization

Crystallization conditions were identified and optimized after screening for crystals using the following screens, Qiagen Cryos, PEGS and Hampton Crystal Screen I. The largest crystals were obtained at 298 K by vapor diffusion in sitting drops by mixing 4 μL of protein solution (15 mg/ml in PBS 7.4.) with 1.5 μL of the reservoir solution (1.6 M ammonium sulfate, 10% (v/v) 1,4-dioxane, and 0.1 M MES pH 6.5) and equilibrating against 350 μL of reservoir solution. Crystals of dimension 0.2 mm X 0.3 mm X 0.5 mm grew after 3 months. The crystallization process was shortened to 5 days by increasing the protein concentration to 50 mg/ml and the temperature to 37 °C, without compromising the diffraction quality of the crystals. A second crystal form was obtained with 100 mm Tris pH 8.5 and 200 mm magnesium chloride as the crystallization buffer. These crystals required higher protein concentrations (150 mg/ml Pry1CAP in 0.1 M Tris pH 8.0) and took up to 3 weeks to grow. No crystallization hits were observed when Pry1CAP was screened in 50 mM sodium acetate pH 4.5, 50 mM sodium HEPES pH 7.5, or sodium citrate pH 6.5, whereas weakly diffracting plate-like crystals were obtained with 50 mM sodium cacodylate as protein storage buffer.

### Data collection, structure determination and analysis

Crystals were transferred into a cryo-protecting solution containing 75% precipitant solution and 25% glycerol for ~30 seconds, and flash-cooled directly in a stream of N_2_ gas at 113 K prior to collecting diffraction data. X-ray diffraction data were collected at the Baylor College of Medicine core facility (Rigaku HTC detector, Rigaku FR-E+ SuperBright microfocus rotating anode generator, with VariMax HF optics) using the Crystal Clear (d*trek) package^35^. Data was integrated using MosFLM and scaled with SCALA^36^. Pry1CAP structures were solved after multiple molecular replacement attempts (MR) using different search models^11^,^12^,^13^ with PHASER^37^,^38^. The optimal model that resulted in the lowest Rfree was a polyalanine model of GAPR-1 (PDB entry 1smb)^14^. The MR solution indicated a monomer per asymmetric unit giving a Matthews’ coefficient of 4.3 Å^3^/Da with 72% solvent content^39^. Molecular replacement was followed by automatic model building using ARP/wARP^40^,^41^. The final models were obtained by iterative manual model building cycles using the program Coot^42^ followed by structure refinement with REFMAC5^43^,^44^ and PHENIX^45^. Structural figures were generated using PyMOL^46^. Structures were analyzed and Fig. 5B was generated using PDBSumm (https://www.ebi.ac.uk/thornton-srv/databases/pdbsum/Generate.html). Details of the quality of the structure as well as data collection are shown in Table 1. Protein structures most similar to Pry1CAP were identified using PDBeFold’s structure similarity option (http://www.ebi.ac.uk/msd-srv/ssm/), which allows a 3-D structural alignment taking both the alignment length and rmsd into account.

### *In vitro* lipid binding

The radioligand binding assay was performed as described previously^25^,^47^. 100 pmol of purified protein in binding buffer (20 mM Tris, pH 7.5, 30 mM NaCl, 0.05% Triton X-100) was incubated with 0–400 pmol of [^3^H]-cholesterol (American Radiolabeled Chemicals Inc., St Louis, Missouri, USA) for 1 h at 30 °C. The protein was then separated from the unbound ligand by adsorption to Q-sepharose beads (GE healthcare, USA), beads were washed, and the radioligand was quantified by scintillation counting. The effect of 1,4-dioxane was determined by performing the *in vitro* assay in the presence of 1,4-dioxane (0–4%v/v). The effect of divalent cations on cholesterol binding was measured by performing the *in vitro* binding reaction in the presence of different concentrations of EDTA and magnesium chloride. At least two independent experiments were performed under each experimental condition and data is reported as the mean ± standard deviation. Calculation of the *K*_*d*_ value and curve fitting was performed using the statistical software GraphPad Prism, La Jolla, CA.

### *In vivo* yeast sterol export assay

Acetylation and export of sterols into the culture supernatant was examined as described elsewhere^26^. In this sterol export assay, the export of radiolabeled cholesterol acetate by *hem1Δ say1Δ* deficient *Saccharomyces cerevisiae* cells upon complementation with plasmid containing genes of interest is monitored. Yeast cells (*say1Δ hem1Δ*) were cultivated in the presence of cholesterol/Tween 80 and were labeled with 0.025 μCi/ml [^14^C]-cholesterol (American Radiolabeled Chemicals Inc., St Louis, Missouri, USA). Cells were harvested by centrifugation, washed twice with synthetic complete (SC) media, and grown overnight in fresh medium containing non-radiolabeled cholesterol. Cells were centrifuged and lipids were extracted from the cell pellet and the culture supernatant using chloroform/methanol [1:1, (v/v)]. Samples were dried and separated by thin-layer chromatography on silica gel 60 plates (TLC; Merck, Darmstadt, Germany) using the solvent system petroleum ether/diethyl ether/acetic acid [70:30:2, (v/v)]. TLCs were exposed to phosphorimager screens and radiolabeled lipids were visualized and quantified using a phosphorimager (Bio-Rad Laboratories, Hercules, California, USA). The export index is the relative levels of CA that is exported by the cells, and calculated as the ratio of extracellular CA to the sum of intracellular and extracellular CA. Export experiments were performed in triplicate and reported as the mean ± standard deviation of three independent experiments.

## Additional Information

**How to cite this article**: Darwiche, R. *et al*. Structural and functional characterization of the CAP domain of pathogen-related yeast 1 (Pry1) protein. *Sci. Rep.* **6**, 28838; doi: 10.1038/srep28838 (2016).

## Supplementary Material

Supplementary Information

## Figures and Tables

**Table 1:** Statistics for data collection and model refinement.

Data Collection	PDB entry 5ete	PDB entry 5jys
X-ray Source	Rigaku FR-E+	Rigaku FR-E+
Detector	Rigaku HTC	Rigaku HTC
Wavelength	0.15418 nm	0.15418 nm
Space group	*P*6_2_22	*I*4_1_
Cell dimensions	a = b = 124.74 Å, c=59.06 Å α = β = 90.00°, γ = 120.00°	a = b = 69.11 Å, c = 97.73 Å α = β = γ = 90.00°
Resolution (Å)	30.8–2.1 (2.16–2.10)	34.5–1.89 (1.99–1.90)
Number of total reflections	230544 (17841)	65957 (4185)
Number of unique reflections	16255 (1304)	18355 (1200)
^*†*^*R*_*merge*_	9.7 (62.4)	8.2 (42.8)
*I/σ*(*I*)	17.5 (3.8)	10.4 (2.3)
Completeness (%)	99.8 (100)	100 (100)
^†^Redundancy	14.2 (13.7)	3.6 (3.5)
Mn(I) half-set correlation CC(1/2)	0.998 (0.963)	0.995 (0.902)
Average Mosaicity	1.8	1.01
Resolution (Å)	30.8–2.01 (2.10–2.01)	34.5–1.79 (1.83–1.79)
Number of total reflections		77208 (4323)
Number of unique reflections	18511 (1324)	21607 (1254)
^*†*^*R*_*merge*_	0.105 (1.296)	0.073 (0.629)
*I/σ*(*I*)	15.4 (1.7)	9.4 (1.4)
Completeness (%)	99.7 (98.9)	100 (100)
Redundancy	14.1 (13.0)	3.6 (3.4)
Refinement (PHENIX)		
Resolution (Å)	28.46–2.10 (2.23–2.10)	34.5–1.9 (2.0–1.9)
Percentage Data completeness	99.4 (99.4)	100 (100)
*R*_*Free*_ test set	831 reflections (5.40%)	961 reflections (5.34%)
Wilson B-factor (Å^2^)	29.5	27.3
Anisotropy	0.171	0.027
No. of non-H protein atoms	1119	1072
No. of water molecules	128	196
Ions and ligands	4 DIO (1,4-dioxane)	1 Mg^2+^
^a^*R*_*work*_	0.167 (0.188)	0.198
^b^*R*_*Free*_	0.190 (0.222)	0.208
Correlation coefficient *F*_*o*_−*F*_*c*_	0.954 (0.939)	0.94
Average B-factors (Å^2^)	31.3	30.2
Protein (Å^2^)	29.2	29.6
Water and other small molecules (Å^2^)	40.5	42.6
r.m.s. deviations		
Bond lengths (Å)	0.011	0.003
Bond angles (^o^)	0.935	0.533
MolProbity analysis		
Ramachandran outliers	0%	0
Ramachandran favored	100%	100
Rotamer outliers	3%	0
C-beta deviations	0	0
Clashscore	0.96	0.99
