# Supplementary material for: Structural and functional characterization of the CAP domain of pathogen-related yeast 1 (Pry1) protein
Source: Sci Rep. 2016 Jun 27;6:28838. doi: 10.1038/srep28838 (PMC4921858; doi:10.1038/srep28838)
Supplement: Supplementary Information [file srep28838-s1.pdf]

Supplementary material for

Structural and functional characterization of the CAP domain of pathogen-related yeast 1 (Pry1) protein

Authors: Rabih Darwiche, Alan Kelleher, Elissa M. Hudspeth, Roger Schneiter, and Oluwatoyin A. Asojo

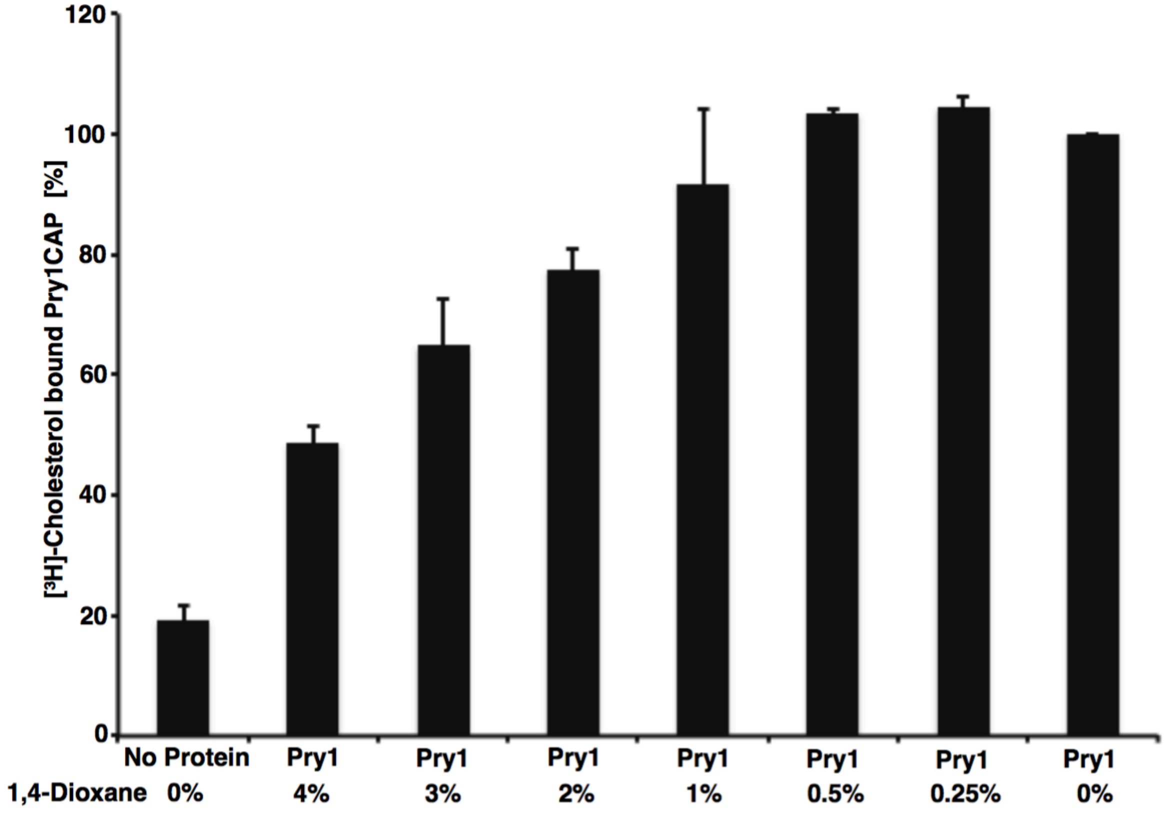

Figure S.1. 1,4-Dioxane inhibits cholesterol binding in a dose dependent manner. Data represents mean ±standard deviation of two independent experiments. In the experiments, purified protein (0 or 100 pmol) in binding buffer (20 mM Tris, pH 7.5, 30 mM NaCl, 0.05% Triton X-100) was incubated with [^{3}H]-cholesterol (50 pmol) with 1,4-dioxane (0-4% v/v) for 1 h at 30°C. The protein was then separated from the unbound ligand by adsorption to Q-sepharose beads (GE healthcare, USA), beads were washed, and the radioligand was quantified by scintillation counting.
